# Dichlorido(di-2-pyridylamine)mercury(II)

**DOI:** 10.1107/S1600536808040294

**Published:** 2008-12-06

**Authors:** Mohammad Yousefi, Mohammad Reza Allahgholi Ghasri, Amene Heidari, Vahid Amani

**Affiliations:** a Islamic Azad University, Shahr-e-Rey Branch, Tehran, Iran; b Department of Chemistry, University of Zabol, Iran

## Abstract

In the mol­ecule of the title compound, [HgCl_2_(C_10_H_9_N_3_)], the Hg^II^ atom is four-coordinated in a distorted tetra­hedral configuration by two N atoms from the chelating di-2-pyridylamine ligand and by two Cl atoms. In the crystal structure, inter­molecular N—H⋯Cl hydrogen bonds link the mol­ecules into centrosymmetric dimers. There is a π–π contact between the pyridine rings [centroid–centroid distance = 3.896 (5) Å].

## Related literature

For related literature, see: Ahmadi *et al.* (2008 ▶); Kalateh, Ebadi *et al.* (2008 ▶); Kalateh, Norouzi *et al.* (2008 ▶); Khavasi *et al.* (2008 ▶); Tadayon Pour *et al.* (2008 ▶); Yousefi, Rashidi Vahid *et al.* (2008 ▶); Yousefi, Tadayon Pour *et al.* (2008 ▶). For related structures, see: Chen *et al.* (2006 ▶); Liu *et al.* (2004 ▶). For bond-length data, see: Allen *et al.* (1987 ▶).
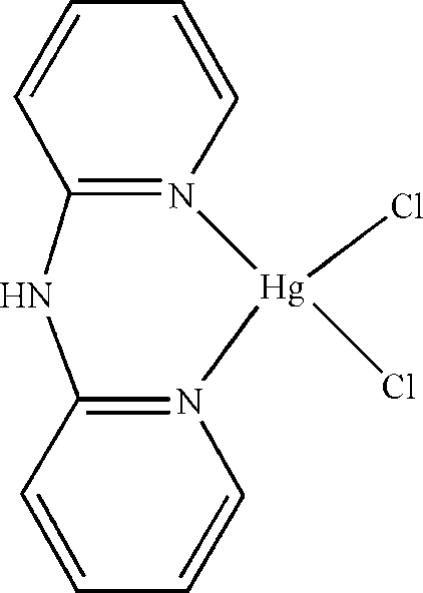


## Experimental

### 

#### Crystal data

[HgCl_2_(C_10_H_9_N_3_)]
*M* *_r_* = 442.69
Triclinic, 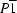

*a* = 8.0268 (12) Å
*b* = 8.6127 (11) Å
*c* = 9.6118 (14) Å
α = 110.606 (11)°
β = 98.958 (12)°
γ = 96.862 (11)°
*V* = 603.38 (15) Å^3^
*Z* = 2
Mo *K*α radiation
μ = 13.17 mm^−1^
*T* = 298 (2) K
0.24 × 0.21 × 0.15 mm

#### Data collection

Bruker SMART CCD area-detector diffractometer
Absorption correction: multi scan (*SADABS*; Sheldrick, 1998 ▶) *T*_min_ = 0.061, *T*_max_ = 0.142
7105 measured reflections
3214 independent reflections
2806 reflections with *I* > 2σ(*I*)
*R*_int_ = 0.069

#### Refinement

*R*[*F* ^2^ > 2σ(*F* ^2^)] = 0.046
*wR*(*F* ^2^) = 0.126
*S* = 1.05
3214 reflections
150 parameters
H atoms treated by a mixture of independent and constrained refinement
Δρ_max_ = 2.43 e Å^−3^
Δρ_min_ = −2.08 e Å^−3^

### 

Data collection: *SMART* (Bruker, 1998 ▶); cell refinement: *SAINT* (Bruker, 1998 ▶); data reduction: *SAINT*; program(s) used to solve structure: *SHELXTL* (Sheldrick, 2008 ▶); program(s) used to refine structure: *SHELXTL*; molecular graphics: *ORTEP-3 for Windows* (Farrugia, 1997 ▶); software used to prepare material for publication: *WinGX* (Farrugia, 1999 ▶).

## Supplementary Material

Crystal structure: contains datablocks I, global. DOI: 10.1107/S1600536808040294/hk2588sup1.cif

Structure factors: contains datablocks I. DOI: 10.1107/S1600536808040294/hk2588Isup2.hkl

Additional supplementary materials:  crystallographic information; 3D view; checkCIF report

## Figures and Tables

**Table:** 

Cl1—Hg1	2.3875 (19)
Cl2—Hg1	2.4579 (19)
N1—Hg1	2.369 (6)
N3—Hg1	2.290 (6)

**Table:** 

N1—Hg1—Cl1	112.30 (15)
N1—Hg1—Cl2	94.92 (15)
N3—Hg1—Cl1	112.21 (15)
N3—Hg1—Cl2	123.92 (14)
N3—Hg1—N1	82.4 (2)
Cl1—Hg1—Cl2	120.14 (7)

**Table 2:** Hydrogen-bond geometry (Å, °)

*D*—H⋯*A*	*D*—H	H⋯*A*	*D*⋯*A*	*D*—H⋯*A*
N2—H2*B*⋯Cl2^i^	0.95 (16)	2.41 (16)	3.345 (6)	169 (13)

Symmetry code: (i) 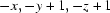.

**Fig. 1.**
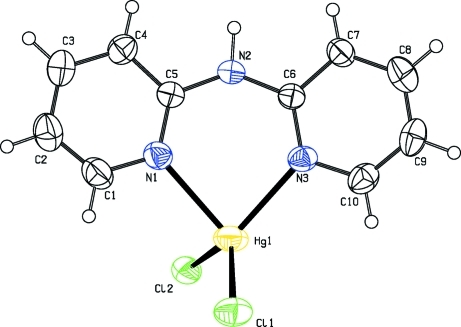
The molecular structure of the title molecule, with the atom-numbering scheme. Displacement ellipsoids are drawn at the 40% probability level.

**Fig. 2.**
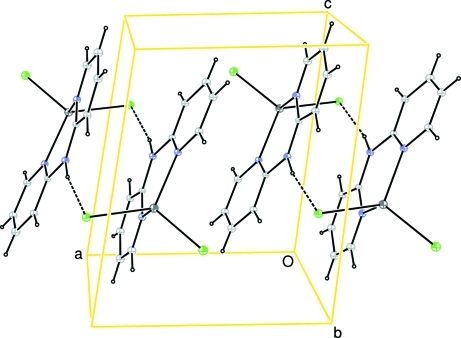
A packing diagram of the title compound. Hydrogen bonds are shown as dashed lines.

## supplementary crystallographic information

### Comment

Recently, we reported the synthes and crystal structures of [Hg(NH(py)_2_)Br_2_], (II), (Kalateh, Norouzi *et al.*, 2008), [Hg(4,4'-dmbpy)I_2_], (III), (Yousefi, Tadayon Pour *et al.*, 2008), [Hg(5,5'-dmbpy)I_2_], (IV), (Tadayon Pour *et al.*, 2008), [Hg(dmphen)I_2_], (V), (Yousefi, Rashidi Vahid *et al.*, 2008), {[HgCl(dm4bt)]_2_(µ-Cl)_2_}, (VI), (Khavasi *et al.*, 2008), [Hg(6-mbpy)Cl_2_], (VII), (Ahmadi *et al.*, 2008) and [{HgBr(4,4'-dmbpy)}_2_(µ-Br)_2_], (VIII), (Kalateh, Ebadi *et al.*, 2008) [where NH(py)_2_ is di-2-pyridylamine, 4,4'-dmbpy is 4,4'-dimethyl-2,2'-bipyridine, 5,5'-dmbpy is 5,5'-dimethyl-2,2'-bipyridine, 6-mbpy is 6-methyl-2,2'-bipyridine, dmphen is 4,7-diphenyl- 1,10-phenanthroline and dm4bt is 2,2'-dimethyl-4,4'-bithiazole].

There are several Hg^II^ complexes, with formula, [Hg(N—N)Cl_2_], such as [Hg(bipy)Cl_2_], (IX), and [Hg(bipy)Cl_2_][HgCl_2_], (X), (Chen *et al.*, 2006) and [Hg(dpdmbip) Cl_2_].CH_2_Cl_2_, (XI), (Liu *et al.*, 2004) [where bipy is 2,2'-bipyridine and dpdmbip is 4,4'-diphenyl-6,6'-dimethyl-2,2'-bipyrimidine] have been synthesized and characterized by single-crystal X-ray diffraction methods. We report herein the synthesis and crystal structure of the title compound, (I).

In the title compound, (Fig. 1), the Hg^II^ atom is four-coordinated in a distorted tetrahedral configuration by two N atoms from di-2-pyridylamine and two Cl atoms. The Hg-Cl and Hg-N bond lengths (Allen *et al.*, 1987) and angles (Table 1) are within normal ranges.

In the crystal structure, intermolecular N-H···Cl hydrogen bonds (Table 2) link the molecules into centrosymmetric dimers, in which they may be effective in the stabilization of the crystal structure (Fig. 2). The π-π contact between the pyridine rings, Cg2···Cg3^i^ [symmetry code: (i) -x, 1 - y, 1 - z, where Cg2 and Cg3 are centroids of the rings A (N1/C1-C5) and B (N3/C6-C10), respectively] may further stabilize the structure, with centroid-centroid distance of 3.896 (5)%A.

### Experimental

For the preparation of the title compound, (I), a solution of di-2-pyridylamine (0.25 g, 1.43 mmol) in methanol (20 ml) was added to a solution of HgCl_2_ (0.39 g, 1.43 mmol) in acetonitrile (20 ml) and the resulting colorless solution was stirred for 20 min at 313 K. This solution was left to evaporate slowly at room temperature. After one week, colorless block crystals of the title compound were isolated (yield; 0.47 g, 74.3%).

### Refinement

H2B atom (for NH) was located in difference synthesis and refined isotropically [N-H = 0.95 (14) Å and U_iso_(H) = 0.10 (4) Å^2^]. The remaining H atoms were positioned geometrically, with C-H = 0.93 Å for aromatic H and constrained to ride on their parent atoms, with U_iso_(H) = 1.2U_eq_(C).

### Crystal data

**Table:**

[HgCl_2_(C_10_H_9_N_3_)]	*Z* = 2
*M**_r_* = 442.69	*F*(000) = 408
Triclinic, *P*1	*D*_x_ = 2.437 Mg m^−^^3^
Hall symbol: -P 1	Mo *K*α radiation, λ = 0.71073 Å
*a* = 8.0268 (12) Å	Cell parameters from 1652 reflections
*b* = 8.6127 (11) Å	θ = 2.6–29.2°
*c* = 9.6118 (14) Å	µ = 13.17 mm^−^^1^
α = 110.606 (11)°	*T* = 298 K
β = 98.958 (12)°	Block, colorless
γ = 96.862 (11)°	0.24 × 0.21 × 0.15 mm
*V* = 603.38 (15) Å^3^	

### Data collection

**Table:**

Bruker SMART CCD area-detector diffractometer	3214 independent reflections
Radiation source: fine-focus sealed tube	2806 reflections with *I* > 2σ(*I*)
graphite	*R*_int_ = 0.069
φ and ω scans	θ_max_ = 29.2°, θ_min_ = 2.6°
Absorption correction: multi scan (*SADABS*; Sheldrick, 1998)	*h* = −10→10
*T*_min_ = 0.061, *T*_max_ = 0.142	*k* = −11→11
7105 measured reflections	*l* = −13→12

### Refinement

**Table:**

Refinement on *F*^2^	Secondary atom site location: difference Fourier map
Least-squares matrix: full	Hydrogen site location: inferred from neighbouring sites
*R*[*F*^2^ > 2σ(*F*^2^)] = 0.046	H atoms treated by a mixture of independent and constrained refinement
*wR*(*F*^2^) = 0.126	*w* = 1/[σ^2^(*F*_o_^2^) + (0.075*P*)^2^ + 0.8992*P*] where *P* = (*F*_o_^2^ + 2*F*_c_^2^)/3
*S* = 1.05	(Δ/σ)_max_ = 0.026
3214 reflections	Δρ_max_ = 2.43 e Å^−^^3^
150 parameters	Δρ_min_ = −2.08 e Å^−^^3^
0 restraints	Extinction correction: *SHELXTL* (Sheldrick, 2008), Fc^*^=kFc[1+0.001xFc^2^λ^3^/sin(2θ)]^-1/4^
Primary atom site location: structure-invariant direct methods	Extinction coefficient: 0.048 (3)

### Special details

**Table:**

Geometry. All e.s.d.'s (except the e.s.d. in the dihedral angle between two l.s. planes) are estimated using the full covariance matrix. The cell e.s.d.'s are taken into account individually in the estimation of e.s.d.'s in distances, angles and torsion angles; correlations between e.s.d.'s in cell parameters are only used when they are defined by crystal symmetry. An approximate (isotropic) treatment of cell e.s.d.'s is used for estimating e.s.d.'s involving l.s. planes.
Refinement. Refinement of *F*^2^ against ALL reflections. The weighted *R*-factor *wR* and goodness of fit *S* are based on *F*^2^, conventional *R*-factors *R* are based on *F*, with *F* set to zero for negative *F*^2^. The threshold expression of *F*^2^ > σ(*F*^2^) is used only for calculating *R*-factors(gt) *etc*. and is not relevant to the choice of reflections for refinement. *R*-factors based on *F*^2^ are statistically about twice as large as those based on *F*, and *R*- factors based on ALL data will be even larger.

### Fractional atomic coordinates and isotropic or equivalent isotropic displacement parameters (Å^2^)

**Table:**

	*x*	*y*	*z*	*U*_iso_*/*U*_eq_	
Hg1	0.21151 (4)	0.24646 (4)	0.63832 (4)	0.06055 (18)	
Cl1	0.4456 (3)	0.1830 (3)	0.7818 (3)	0.0683 (5)	
Cl2	−0.0856 (2)	0.1450 (2)	0.6448 (2)	0.0553 (4)	
N1	0.1944 (8)	0.5364 (8)	0.7382 (7)	0.0497 (12)	
N2	0.2047 (8)	0.5833 (7)	0.5110 (6)	0.0459 (11)	
N3	0.2876 (7)	0.3143 (7)	0.4439 (7)	0.0447 (11)	
C1	0.1758 (12)	0.5982 (12)	0.8836 (9)	0.0636 (19)	
H1	0.1862	0.5294	0.9393	0.076*	
C2	0.1428 (12)	0.7553 (13)	0.9537 (9)	0.069 (2)	
H2	0.1285	0.7920	1.0537	0.083*	
H2B	0.186 (18)	0.662 (18)	0.464 (16)	0.10 (4)*	
C3	0.1312 (11)	0.8591 (11)	0.8713 (9)	0.0624 (18)	
H3	0.1115	0.9683	0.9157	0.075*	
C4	0.1493 (9)	0.7975 (9)	0.7231 (8)	0.0514 (14)	
H4	0.1379	0.8635	0.6649	0.062*	
C5	0.1848 (7)	0.6352 (7)	0.6597 (7)	0.0398 (11)	
C6	0.2674 (7)	0.4531 (7)	0.4151 (7)	0.0395 (10)	
C7	0.3045 (9)	0.4711 (10)	0.2836 (7)	0.0499 (13)	
H7	0.2849	0.5656	0.2627	0.060*	
C8	0.3715 (12)	0.3449 (13)	0.1842 (10)	0.067 (2)	
H8	0.4006	0.3559	0.0977	0.080*	
C9	0.3935 (9)	0.2066 (10)	0.2156 (9)	0.0587 (18)	
H9	0.4365	0.1208	0.1498	0.070*	
C10	0.3524 (9)	0.1931 (9)	0.3444 (10)	0.0556 (16)	
H10	0.3694	0.0976	0.3648	0.067*	

### Atomic displacement parameters (Å^2^)

**Table:**

	*U*^11^	*U*^22^	*U*^33^	*U*^12^	*U*^13^	*U*^23^
Hg1	0.0618 (2)	0.0660 (2)	0.0781 (3)	0.02555 (14)	0.02487 (14)	0.04774 (18)
Cl1	0.0653 (11)	0.0710 (11)	0.0816 (13)	0.0210 (9)	0.0091 (9)	0.0445 (10)
Cl2	0.0623 (9)	0.0551 (8)	0.0628 (9)	0.0170 (7)	0.0277 (7)	0.0314 (7)
N1	0.051 (3)	0.058 (3)	0.050 (3)	0.019 (2)	0.014 (2)	0.027 (2)
N2	0.059 (3)	0.043 (2)	0.045 (3)	0.016 (2)	0.015 (2)	0.024 (2)
N3	0.043 (2)	0.041 (2)	0.056 (3)	0.006 (2)	0.013 (2)	0.023 (2)
C1	0.072 (5)	0.078 (5)	0.051 (4)	0.020 (4)	0.017 (3)	0.033 (4)
C2	0.071 (5)	0.084 (6)	0.047 (4)	0.019 (4)	0.012 (3)	0.017 (4)
C3	0.066 (4)	0.061 (4)	0.052 (4)	0.013 (4)	0.015 (3)	0.009 (3)
C4	0.053 (3)	0.045 (3)	0.052 (3)	0.007 (3)	0.008 (3)	0.014 (3)
C5	0.035 (2)	0.043 (3)	0.043 (3)	0.006 (2)	0.007 (2)	0.019 (2)
C6	0.037 (2)	0.040 (3)	0.042 (3)	0.005 (2)	0.008 (2)	0.017 (2)
C7	0.052 (3)	0.060 (4)	0.046 (3)	0.017 (3)	0.015 (2)	0.026 (3)
C8	0.064 (4)	0.088 (6)	0.057 (4)	0.013 (4)	0.025 (3)	0.032 (4)
C9	0.045 (3)	0.054 (4)	0.063 (4)	0.008 (3)	0.018 (3)	0.003 (3)
C10	0.049 (3)	0.047 (3)	0.072 (4)	0.011 (3)	0.020 (3)	0.021 (3)

### Geometric parameters (Å, °)

**Table:**

Cl1—Hg1	2.3875 (19)	C5—N1	1.323 (8)
Cl2—Hg1	2.4579 (19)	C5—N2	1.381 (8)
N1—Hg1	2.369 (6)	C6—N3	1.341 (8)
N2—H2B	0.95 (14)	C6—N2	1.383 (8)
N3—Hg1	2.290 (6)	C6—C7	1.398 (9)
C1—N1	1.349 (10)	C7—C8	1.397 (11)
C1—C2	1.363 (13)	C7—H7	0.9300
C1—H1	0.9300	C8—C9	1.352 (14)
C2—C3	1.390 (14)	C8—H8	0.9300
C2—H2	0.9300	C9—C10	1.369 (12)
C3—C4	1.372 (11)	C9—H9	0.9300
C3—H3	0.9300	C10—N3	1.362 (9)
C4—C5	1.399 (9)	C10—H10	0.9300
C4—H4	0.9300		
			
N1—Hg1—Cl1	112.30 (15)	N2—C6—C7	116.6 (5)
N1—Hg1—Cl2	94.92 (15)	C8—C7—C6	119.0 (7)
N3—Hg1—Cl1	112.21 (15)	C8—C7—H7	120.5
N3—Hg1—Cl2	123.92 (14)	C6—C7—H7	120.5
N3—Hg1—N1	82.4 (2)	C9—C8—C7	119.0 (7)
Cl1—Hg1—Cl2	120.14 (7)	C9—C8—H8	120.6
N1—C1—C2	123.8 (8)	C7—C8—H8	120.4
N1—C1—H1	118.1	C8—C9—C10	119.9 (7)
C2—C1—H1	118.1	C8—C9—H9	120.0
C1—C2—C3	117.9 (8)	C10—C9—H9	120.1
C1—C2—H2	121.0	N3—C10—C9	122.5 (7)
C3—C2—H2	121.0	N3—C10—H10	118.7
C4—C3—C2	118.7 (8)	C9—C10—H10	118.8
C4—C3—H3	120.6	C5—N1—C1	118.4 (7)
C2—C3—H3	120.7	C5—N1—Hg1	125.5 (4)
C3—C4—C5	119.9 (7)	C1—N1—Hg1	115.7 (5)
C3—C4—H4	120.1	C6—N2—C5	136.0 (5)
C5—C4—H4	120.0	C6—N2—H2B	109 (8)
N1—C5—N2	122.3 (6)	C5—N2—H2B	115 (8)
N1—C5—C4	121.1 (6)	C6—N3—C10	118.2 (6)
N2—C5—C4	116.6 (6)	C6—N3—Hg1	127.3 (4)
N3—C6—N2	122.1 (6)	C10—N3—Hg1	114.4 (5)
N3—C6—C7	121.3 (6)		
			
C1—N1—Hg1—N3	−169.7 (6)	C6—C7—C8—C9	2.0 (12)
C5—N1—Hg1—N3	17.3 (5)	C7—C8—C9—C10	−0.9 (13)
C1—N1—Hg1—Cl1	−58.7 (6)	C8—C9—C10—N3	0.6 (12)
C1—N1—Hg1—Cl2	66.7 (6)	N2—C5—N1—C1	179.1 (7)
C5—N1—Hg1—Cl1	128.2 (5)	C4—C5—N1—C1	−2.6 (10)
C5—N1—Hg1—Cl2	−106.3 (5)	N2—C5—N1—Hg1	−8.1 (9)
C6—N3—Hg1—N1	−15.5 (5)	C4—C5—N1—Hg1	170.3 (5)
C10—N3—Hg1—N1	168.0 (5)	C2—C1—N1—C5	1.9 (13)
C6—N3—Hg1—Cl1	−126.5 (5)	C2—C1—N1—Hg1	−171.6 (7)
C10—N3—Hg1—Cl1	57.0 (5)	N3—C6—N2—C5	17.7 (11)
C6—N3—Hg1—Cl2	75.3 (5)	C7—C6—N2—C5	−164.1 (7)
C10—N3—Hg1—Cl2	−101.2 (5)	N1—C5—N2—C6	−15.3 (11)
N1—C1—C2—C3	−1.4 (15)	C4—C5—N2—C6	166.3 (7)
C1—C2—C3—C4	1.5 (14)	N2—C6—N3—C10	−179.2 (6)
C2—C3—C4—C5	−2.3 (12)	C7—C6—N3—C10	2.7 (9)
C3—C4—C5—N1	2.8 (10)	N2—C6—N3—Hg1	4.5 (8)
C3—C4—C5—N2	−178.7 (7)	C7—C6—N3—Hg1	−173.7 (5)
N3—C6—C7—C8	−3.0 (10)	C9—C10—N3—C6	−1.5 (10)
N2—C6—C7—C8	178.7 (7)	C9—C10—N3—Hg1	175.3 (6)

### Hydrogen-bond geometry (Å, °)

**Table:**

*D*—H···*A*	*D*—H	H···*A*	*D*···*A*	*D*—H···*A*
N2—H2B···Cl2^i^	0.95 (16)	2.41 (16)	3.345 (6)	169 (13)

Symmetry codes: (i) −*x*, −*y*+1, −*z*+1.

## References

[bb1] Ahmadi, R., Ebadi, A., Kalateh, K., Norouzi, A. & Amani, V. (2008). *Acta Cryst.* E**64**, m1407. 10.1107/S1600536808032777 PMC2959542 21580857

[bb2] Allen, F. H., Kennard, O., Watson, D. G., Brammer, L., Orpen, A. G. & Taylor, R. (1987). *J. Chem. Soc. Perkin Trans. 2*, pp. S1–19.

[bb3] Bruker (1998). *SMART* and *SAINT* Bruker AXS Inc., Madison, Wisconsin, USA.

[bb4] Chen, W. T., Wang, M. S., Liu, X., Guo, G. C. & Huang, J. S. (2006). *Cryst. Growth Des.* **6**, 2289–2300.

[bb5] Farrugia, L. J. (1997). *J. Appl. Cryst.* **30**, 565.

[bb6] Farrugia, L. J. (1999). *J. Appl. Cryst.* **32**, 837–838.

[bb7] Kalateh, K., Ebadi, A., Ahmadi, R., Amani, V. & Khavasi, H. R. (2008). *Acta Cryst.* E**64**, m1397–m1398. 10.1107/S1600536808032510 PMC2959555 21580848

[bb8] Kalateh, K., Norouzi, A., Ebadi, A., Ahmadi, R. & Amani, V. (2008). *Acta Cryst.* E**64**, m1583–m1584. 10.1107/S1600536808038129 PMC2960074 21581184

[bb9] Khavasi, H. R., Abedi, A., Amani, V., Notash, B. & Safari, N. (2008). *Polyhedron*, **27**, 1848–1854.

[bb10] Liu, Q. D., Wang, R. & Wang, S. (2004). *Dalton Trans.* pp. 2073–2079. 10.1039/B404905E 15249941

[bb11] Sheldrick, G. M. (1998). *SADABS* Bruker AXS Inc., Madison, Wisconsin, USA.

[bb12] Sheldrick, G. M. (2008). *Acta Cryst.* A**64**, 112–122. 10.1107/S0108767307043930 18156677

[bb13] Tadayon Pour, N., Ebadi, A., Abedi, A., Amani, V. & Khavasi, H. R. (2008). *Acta Cryst.* E**64**, m1305. 10.1107/S160053680802953X PMC2959332 21201044

[bb14] Yousefi, M., Rashidi Vahid, R., Amani, V., Arab Chamjangali, M. & Khavasi, H. R. (2008). *Acta Cryst.* E**64**, m1339–m1340. 10.1107/S160053680803081X PMC2959289 21201072

[bb15] Yousefi, M., Tadayon Pour, N., Amani, V. & Khavasi, H. R. (2008). *Acta Cryst.* E**64**, m1259. 10.1107/S1600536808028791 PMC2959485 21201014

